# Interleukin-25 Produced by Synoviocytes Has Anti-inflammatory Effects by Acting As a Receptor Antagonist for Interleukin-17A Function

**DOI:** 10.3389/fimmu.2017.00647

**Published:** 2017-05-31

**Authors:** Fabien Lavocat, Ndiémé Ndongo-Thiam, Pierre Miossec

**Affiliations:** ^1^Department of Immunology and Rheumatology, Immunogenomics and Inflammation Research Unit EA 4130, University of Lyon, Edouard Herriot Hospital, Lyon, France

**Keywords:** rheumatoid arthritis, interleukin-17A, interleukin-25, synoviocytes, interleukin-17 receptors

## Abstract

The production and function of cytokines are highly regulated. One mechanism is the balance between pro- and anti-inflammatory cytokines. As interleukin (IL)-17A and IL-25 share the IL-17RA receptor chain, we hypothesize that IL-25 acts as an IL-17A receptor antagonist and limits its pro-inflammatory effects. The production and expression kinetics of IL-25 and its receptor chains IL-17RA and RB were analyzed in rheumatoid synoviocytes alone or in coculture with peripheral blood mononuclear cells (PBMCs). The effects of autocrine or exogenous IL-25 on synoviocytes were investigated in the presence or not of an anti-IL-25 antibody. To study the regulatory effects of IL-25, synoviocytes and/or PBMCs were exposed to IL-25 before being treated with IL-17A and tumor necrosis factor alpha (TNF-α) alone or combined. IL-25, IL-6, and bioactive IL-17A were quantified in rheumatoid arthritis (RA) patient plasma. Synoviocytes expressed and secreted IL-25, and expressed the two chains of its receptor IL-17RA and IL-17RB. IL-17RB expression was increased by TNF-α. IL-25 production occurred at a delayed time point (5 days) after stimulation with IL-17A and TNF-α. Synoviocytes pretreated with IL-25 were less responsive to IL-17A and TNF-α. PBMCs exposed to IL-25 showed a decreased production of pro-inflammatory mediators, including IL-17A with a 57% decrease; *p* = 0.002. IL-25 levels were elevated in the plasma of RA patients compared to healthy subjects (*p* = 0.03). However, these levels are not high enough to inhibit the function of circulating IL-17A. In conclusion, it was shown for the first time that synoviocytes produce IL-25, specifically at late time points and that IL-25 acts as a regulator of IL-17A-driven inflammation, as indicated by *in vitro* results and *in vivo*, in a long-term RA patient follow-up. These results may be important when considering IL-17A inhibition.

## Introduction

The expression and function of cytokines are highly regulated through a large variety of mechanisms. For example, interleukin (IL)-1 is controlled by the IL-1 receptor antagonist (IL-1Ra), which binds to IL-1 receptors and competitively antagonizes the binding of IL-1, thereby reducing its biological effects (1). *In vitro* and *in vivo* increased production of IL-1 is followed by a delayed production of IL-1Ra, acting as a delayed regulatory mechanism.

Interleukin-17A is the prototype cytokine of the IL-17 family, which consists of six members, from IL-17A to IL-17F. IL-17A and IL-17F share 50% sequence homology and are involved in chronic inflammation and autoimmunity (2, 3). IL-25 (also known as IL-17E) shares only 17% sequence homology with IL-17A. Unlike other members of the family, it is involved in the regulation of type-2 immune response, including host defense against parasites (4) and allergy (5, 6). In addition, IL-25 regulates inflammation by controlling the Th17 response (7–10).

Interleukin-17-receptor family comprises five members (IL-17RA to IL-17RE). IL-17 family signal is transduced through a heterodimeric receptor complex consisting of the IL-17RA and IL-17RC chains for IL-17A and IL-17F (11), and IL-17RA and IL-17RB chains for IL-25 (12). Thus, despite their opposite biological effects, IL-17A and IL-25 share the common receptor chain IL-17RA.

In the present study, it was hypothesized that IL-25 could have the same effect on IL-17A signal than IL-1Ra on that of IL-1. As IL-25 and IL-17A share the IL-17RA receptor chain, the presence of IL-25 in the medium could reduce the quantity of available IL17RA chains. This would result in an inhibition of IL-17A biological effects. This aspect is important when testing the effect of IL-17A or IL-17F inhibitors vs. that of IL-17RA. This was studied in rheumatoid arthritis (RA) synoviocytes stimulated with IL-17A and tumor necrosis factor alpha (TNF-α), which act synergistically to induce a massive inflammatory signal (13). The effect of IL-25 was already studied in both *in vitro* and animal models of RA but mainly through its action on T-cells (10). Here, the regulatory effects of IL-25 were investigated in synoviocytes, which are critically involved in RA pathogenesis and perpetuation (14).

## Materials and Methods

### Cell Culture and Experimental Design

Synoviocytes were obtained from the synovial tissue from RA patients undergoing knee or hip surgery. The RA patients fulfilled the American College of Rheumatology criteria of RA (15). Each individual signed an informed consent, and the protocol was approved by the committee for protection of persons participating in biomedical research under the number AC-2010-11-64. Cells were cultured at 37°C/5% CO_2_ in DMEM medium (Eurobio, Courtaboeuf, France) supplemented with 10% fetal bovine serum (Life Technologies, Carlsbad, CA, USA), 2% penicillin–streptomycin (Eurobio, Courtaboeuf, France), 1% l-glutamine (Eurobio, Courtaboeuf, France), and 1% amphotericin B (Eurobio, Courtaboeuf, France). For cytokine treatments, cells were plated at a density of 5 × 10^4^ cells/cm^2^ and left for adhesion overnight before the addition of 50 ng/mL recombinant human IL-17A, 0.5 ng/mL recombinant human TNF-α and/or 50 ng/mL recombinant human IL-25 (all from R&D Systems, Minneapolis, MN, USA).

For IL-25 blocking experiments, synoviocyte supernatants from late time points were incubated for 1 h at 37°C in the presence of an anti-IL-25 polyclonal antibody 10 µg/mL or of a polyclonal goat control IgG (all from R&D Systems, Minneapolis, MN, USA) at 10 µg/mL. Synoviocytes were then pretreated 4 h with supernatants at a final concentration of 5%, then stimulated with 50 ng/mL IL-17A and 0.5 ng/mL TNF-α.

For coculture experiments, peripheral blood mononuclear cells (PBMCs) were obtained from healthy blood donors and isolated by Ficoll-Hypaque (1.077 g/mL) density gradient centrifugation. PBMCs were activated or not with 5 µg/mL anti-CD3 plus 5 µg/mL anti-CD28 monoclonal antibodies (Beckman-Coulter, Brea, CA, USA) and added on adherent synoviocytes at a ratio of five PBMCs for one synoviocyte with or without 50 ng/mL IL-25 (R&D Systems, Minneapolis, MN, USA).

### Quantitative RT-PCR Analysis

RNA was purified using RNeasy kits (Qiagen, Hilden, Germany). Total RNA was quantified by a Qubit^®^ fluorometer using the Qubit^®^ RNA BR assay kit (Life Technologies, Carlsbad, CA, USA). RNA was reverse-transcribed with the QuantiTect Reverse Transcription Kit (Qiagen, Hilden, Germany) and PCR amplification was performed on a CFX96 Real-time PCR Detection System (BioRad, Hercules, CA, USA) using the QuantiFast SYBR Green PCR Kit (Qiagen, Hilden, Germany). The expression of the genes was normalized to the expression of GAPDH.

### Enzyme-Linked Immunosorbent Assay (ELISA)

Interleukin-6, IL-17A, and chemokine (C-C motif) ligand 20 (CCL20) productions were measured in supernatants with commercially available ELISA kits (R&D Systems, Minneapolis, MN, USA), according to the manufacturer’s instructions. IL-25 was measured in cell culture supernatants and in human plasma using IL-25 ELISA kit (PeproTech, Rocky Hill, NJ, USA) according to the manufacturer’s instructions.

### siRNA Transfection

A mixture of four siRNA duplexes (siGENOME SMARTPool siRNA) specific for IL17RA (NM_014339), IL17RB (NM_018725) and IL17RC (NM_032732) were purchased from Dharmacon (Lafayette, LA, USA). RA synoviocytes were used at 80–90% confluence. Cells were transfected with control siRNAs (siGENOME Non-Targeting Control siRNA #4) or target siRNAs by nucleofection using Amaxa technology (Lonza, Basel, Switzerland) according to the manufacturer’s instructions (program U23; Human Dermal Fibroblast Nucleofector kit). Dose- and time-response experiments were performed to determine the best time point and the lowest suitable concentration of siRNA duplexes needed for efficacious RNA silencing. Thus, 5 × 10^5^ cells were nucleofected with 0.5 µg of IL17RA, IL17RB, or IL17RC siRNA duplexes alone or in combination. Forty-eight hours post-transfection, part of the cells was stimulated as explained above in the Section “Cell Culture and Experimental Design” and another part was dedicated to siRNA efficacy control by qRT-PCR analysis of IL17RA, IL17RB, and IL17RC gene expression.

### Bioassay for Circulating Bioactive IL-17A

As previously described (16), synoviocytes from RA patients were cultured in 96-well plates at a density of 1 × 10^4^ cells/well in complete DMEM at 37°C/5% CO_2_ overnight. Cells were treated with heat-decomplemented plasma from RA patients diluted at 10% in serum-free DMEM for 24 h. To evaluate the activity related to circulating IL-17A, an anti-IL-17A antibody (R&D Systems, Minneapolis, MN, USA) at 10 µg/mL was incubated with plasma for 1 h before the addition to synoviocytes. Recombinant IL-17A (R&D Systems, Minneapolis, MN, USA) was used at 50 ng/mL as a positive control. After 48 h, supernatants were collected and IL-6 was measured by ELISA. IL-17A pro-inflammatory dependent level or delta IL-6 production (nanograms per milliliter) represents the production of IL-6 with and without addition of anti-IL-17A antibody.

## Results

### Synoviocytes Produce IL-25 at Late Time Points

The IL-25 expression and production were analyzed over time in RA synoviocytes treated or not with IL-17A and TNF-α. At the same time, IL-6 expression and production were quantified to reflect the level of inflammation.

**Figure 1:**
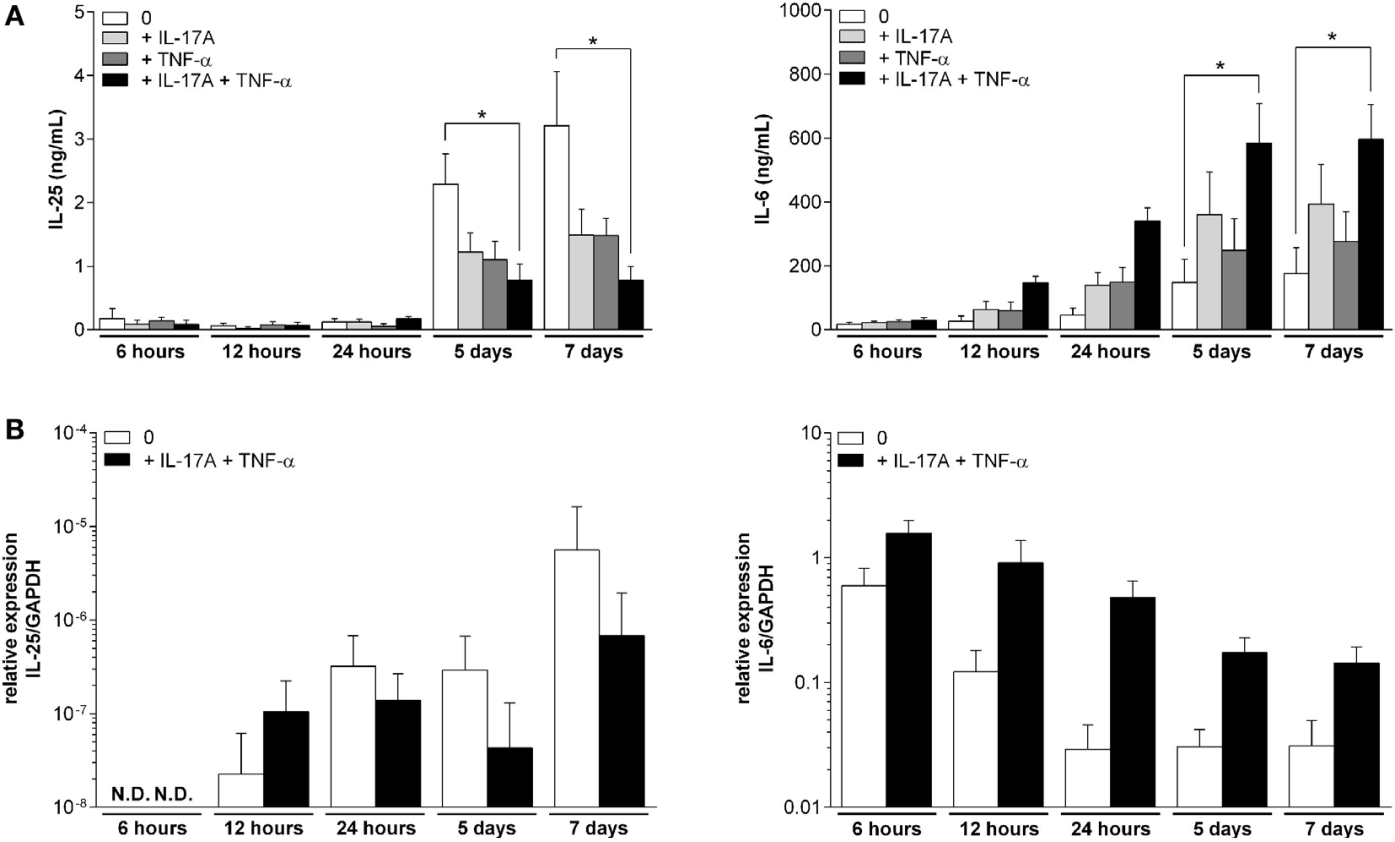
Kinetics of the production and expression of interleukin (IL)-25 and IL-6 in synoviocytes. Cells were treated for 7 days with IL-17A (50 ng/mL) and/or tumor necrosis factor alpha (0.5 ng/mL). At 6, 12, and 24 h and 5 and 7 days, supernatants were collected and RNA was extracted. IL-25 and IL-6 production were quantified by enzyme-linked immunosorbent assay **(A)**. IL-25 and IL-6 expression were quantified by qRT-PCR **(B)**. *p < 0.05 (Mann–Whitney non-parametric test).

In unstimulated synoviocytes, IL-25 production remained low during the first 24 h and started increasing at day 5 (19-fold increase in IL-25 production between day 1 and 5, Figure 1A). This production was significantly reduced after treatment with IL-17A and TNF-α combination (*p* < 0.05, at day 5 and 7). On the contrary, IL-6 production kept increasing in the medium until day 5 and then remained stable. In cells treated with both IL-17A and TNF-α, IL-6 production increased mainly during day 1, as almost 60% of the final IL-6 quantity was already produced after 24 h. As expected, IL-6 production was significantly higher in cells stimulated with a combination of IL-17A and TNF-α when compared to control cells (*p* < 0.05 at day 5 and day 7).

At the mRNA level, IL-25 expression was very low and even undetectable at 6 h (Figure 1B). In unstimulated synoviocytes, IL-25 expression was detectable after 12 h and reached its maximum at day 7, being 250-fold more expressed compared to the 12-h level. In IL-17A and TNF-α-stimulated synoviocytes, IL-25 expression was higher at 12 h compared to resting cells but it increased more slowly (only 6.5-fold increase between 12 h and day 7), a first indication of the inhibitory effect of IL-17A and TNF-α on IL-25 production. In parallel, the highest IL-6 expression was observed at 6 h both in unstimulated or stimulated synoviocytes. Then, IL-6 expression decreased over time, with an early decrease during the first 24 h in unstimulated synoviocytes and a later decrease in stimulated synoviocytes.

These results suggested that IL-25 expression and production occurred at a late time point, i.e., mostly after 5 days of culture, whereas IL-6 production level in response to IL-17A and TNF-α combination strongly increased during the first 24 h and then stabilized as its expression decreased.

### IL-25 Pretreatment Inhibits IL-17A Signaling in Synoviocytes

**Figure 2:**
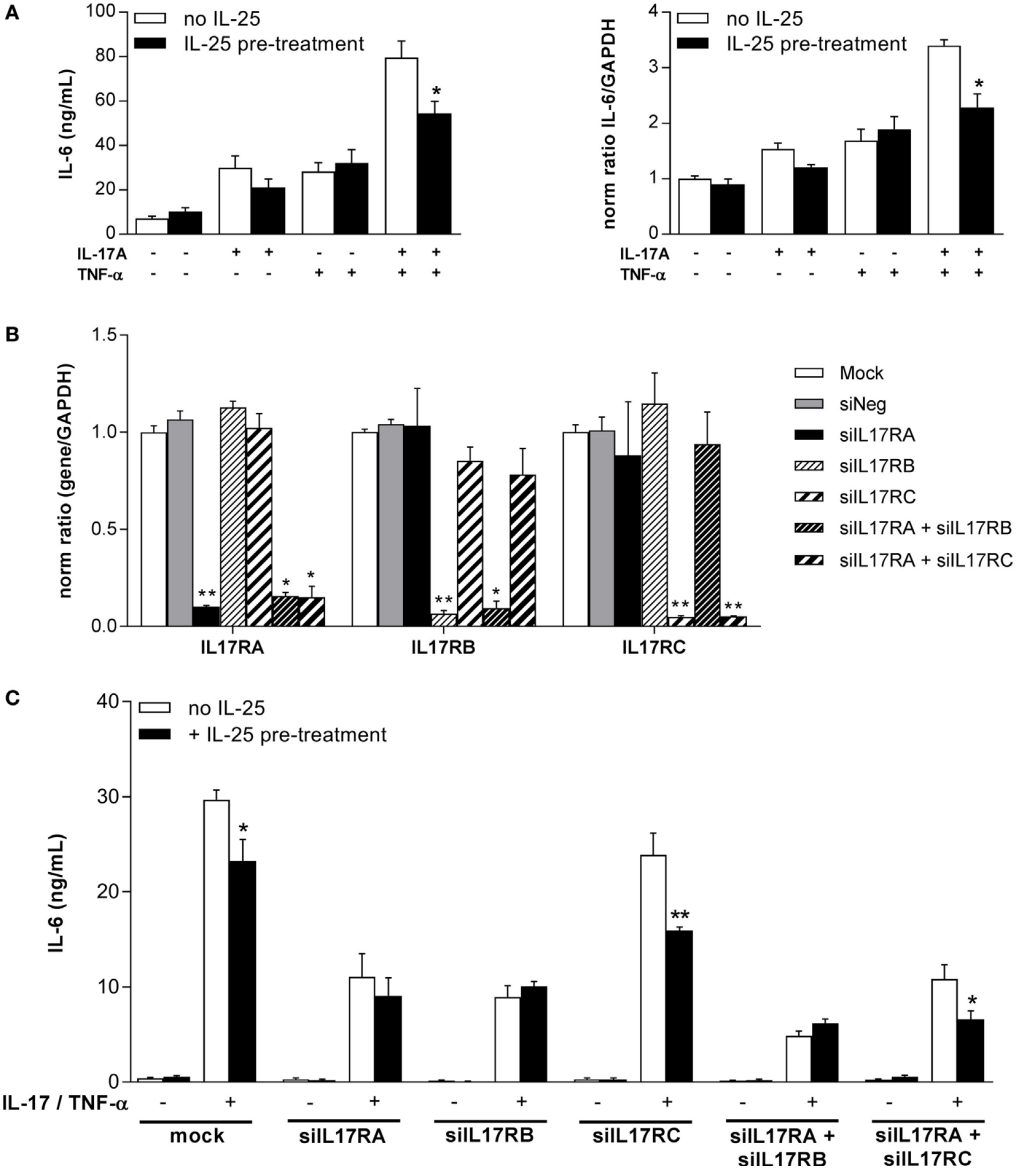
Effects of an interleukin (IL)-25 pretreatment on synoviocyte IL-6 expression and production. **(A)** Cells were pretreated for 4 h with 50 ng/mL IL-25 and then treated with IL-17A (50 ng/mL) and/or tumor necrosis factor alpha (TNF-α) (0.5 ng/mL). RNA was extracted after 12 h and supernatants were collected after 24 h. The production of IL-6 was quantified by enzyme-linked immunosorbent assay (ELISA) and the expression of IL-6 was assessed by qRT-PCR. *p < 0.05 (Mann–Whitney non-parametric test). **(B)** Cells were transfected with 0.5 µg siRNAs targeting IL17RA, IL17RB, and IL17RC alone or in combination. After 48 h, RNA was extracted and the expression of IL17RA, IL17RB, and IL17RC was analyzed by qRT-PCR. **p < 0.01 and *p < 0.05 (Mann–Whitney non-parametric test). **(C)** Cells were transfected with 0.5 µg siRNAs targeting IL17RA, IL17RB, and IL17RC alone or in combination. After 48 h, cells were pretreated for 4 h with 50 ng/mL IL-25 and then treated with IL-17A (50 ng/mL) and TNF-α (0.5 ng/mL). The production of IL-6 was quantified by ELISA. **p < 0.01 and *p < 0.05 (Mann–Whitney non-parametric test).

To investigate whether IL-25 could inhibit IL-17A function from interactions at the receptor level, synoviocytes were pretreated for 4 h with IL-25 before the addition of IL-17A and/or TNF-α. The expression and production of IL-6 were then measured (Figure 2A). A slight but not significant reduction of IL-17A-induced IL-6 expression and production was observed following pretreatment with IL-25. However, a significant decrease was observed with the combination of IL-17A and TNF-α (54.5 ± 17 ng/mL with IL-25 pretreatment vs. 79.6 ± 26 ng/mL without, *p* = 0.02). The same effect was observed for IL-6 expression (2.3-fold increase with IL-25 pretreatment vs. 3.4-fold increase without IL-25, *p* = 0.03).

### Silencing of IL-17R Family Members Disturbs Response to Cytokines

To better understand the role of IL17RA, IL17RB, and IL17RC in the response of synoviocytes to IL-17A and IL-25, a siRNA strategy was set up. Synoviocytes were transfected with siRNA targeting IL17RA, IL17RB, and IL17RC either alone or in combination (combination of IL17RA and IL17RB, for the IL-25 receptor, or combination of IL17RA and IL17RC, for the IL-17A and F receptor). After control of the efficacy of silencing (Figure 2B), cells were pretreated with IL-25 for 4 h before being stimulated with IL-17A and TNF-α (Figure 2C).

As shown in Figure 2B, siRNAs were efficient to inhibit the expression of IL17RA (90% inhibition for siIL17RA alone, 84% inhibition for the siIL17RA and siIL17RB combination, and 85% inhibition for the siIL17RA and siIL17RC combination), IL17RB (93% inhibition for siIL17RB alone and 91% inhibition for the siIL17RA and IL17RB combination) and IL17RC (95% inhibition both for siIL17RC alone or in combination with siIL17RA). The specificity of siRNA was also satisfactory as for instance, siIL17RA had no effect on the expression of IL17RB or IL17RC.

The effect of IL-25 pretreatment on IL-6 production after siRNA transfection was summarized in Figure 2C. In control condition, IL-25 pretreatment inhibited the effects of IL-17A and TNF-α combination, as expected. IL17RA silencing induced a decrease in IL-6 production following treatment with IL-17A and TNF-α and almost no effect of IL-25 pretreatment was observed anymore. This was also expected as both IL-17A and IL-25 transduce their signal through IL17RA. Unexpectedly, silencing of IL17RB had quite the same effect than IL17RA silencing, with a decreased IL-6 production and no effect of IL-25 anymore. However, silencing of IL17RC induced only a weak decrease of IL-6 production and the effect of IL-25 pretreatment was still observed. The combination of IL17RA and IL17RB silencing decreased IL-6 production following IL-17A and TNF-α stimulation even more than after IL17RA silencing alone. Here again, no effect of IL-25 was observed any more. Finally, the combination of IL17RA and IL17RC silencing showed a decreased IL-6 production with a reduced effect of IL-25 pretreatment.

### IL-25 Produced by Synoviocytes Is Functional and Can Inhibit IL-17A Signal

Synoviocyte supernatants were harvested at day 7, when IL-25 production was at the highest level, and were used to assess the bioactivity of IL-25. Synoviocytes were pretreated for 4 h with those supernatants before addition of IL-17A and TNF-α. Before pretreatment, supernatants were incubated with a control antibody or an anti-IL-25 antibody for 1 h to inhibit the effect of IL-25 present in the supernatants.

**Figure 3:**
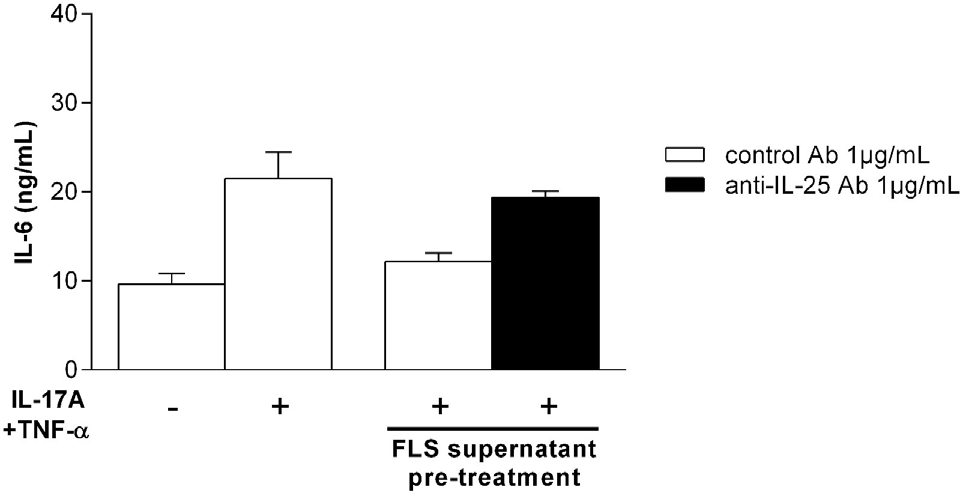
Autocrine effect of synoviocyte-derived interleukin (IL)-25 on IL-6 production. Synoviocytes were pretreated for 4 h with day 7 synoviocyte supernatants (5%) containing autocrine IL-25. They were then treated with IL-17A (50 ng/mL) and tumor necrosis factor alpha (0.5 ng/mL) to induce IL-6 production in the presence of a control antibody (1 µg/mL) or an anti-IL-25 antibody (1 µg/mL). IL-6 production was quantified in supernatants at 24 h by enzyme-linked immunosorbent assay.

As shown in Figure 3, the addition of IL-17A and TNF-α induced an increase in IL-6 production (9.6 ± 1.2 ng/mL in control condition vs. 21.5 ± 5.2 ng/mL after cytokine treatment). Then, as observed with recombinant IL-25, pretreatment with late synoviocyte supernatants, containing high levels of synoviocyte-derived IL-25, reduced by 45% the IL-17A and TNF-α effect on IL-6 production. Conversely, the addition of an anti-IL-25 antibody restored the pro-inflammatory effect of IL-17A and TNF-α.

### Synoviocytes Express IL-17 Family Receptors and IL-17RB Is Regulated by TNF-α

**Figure 4:**
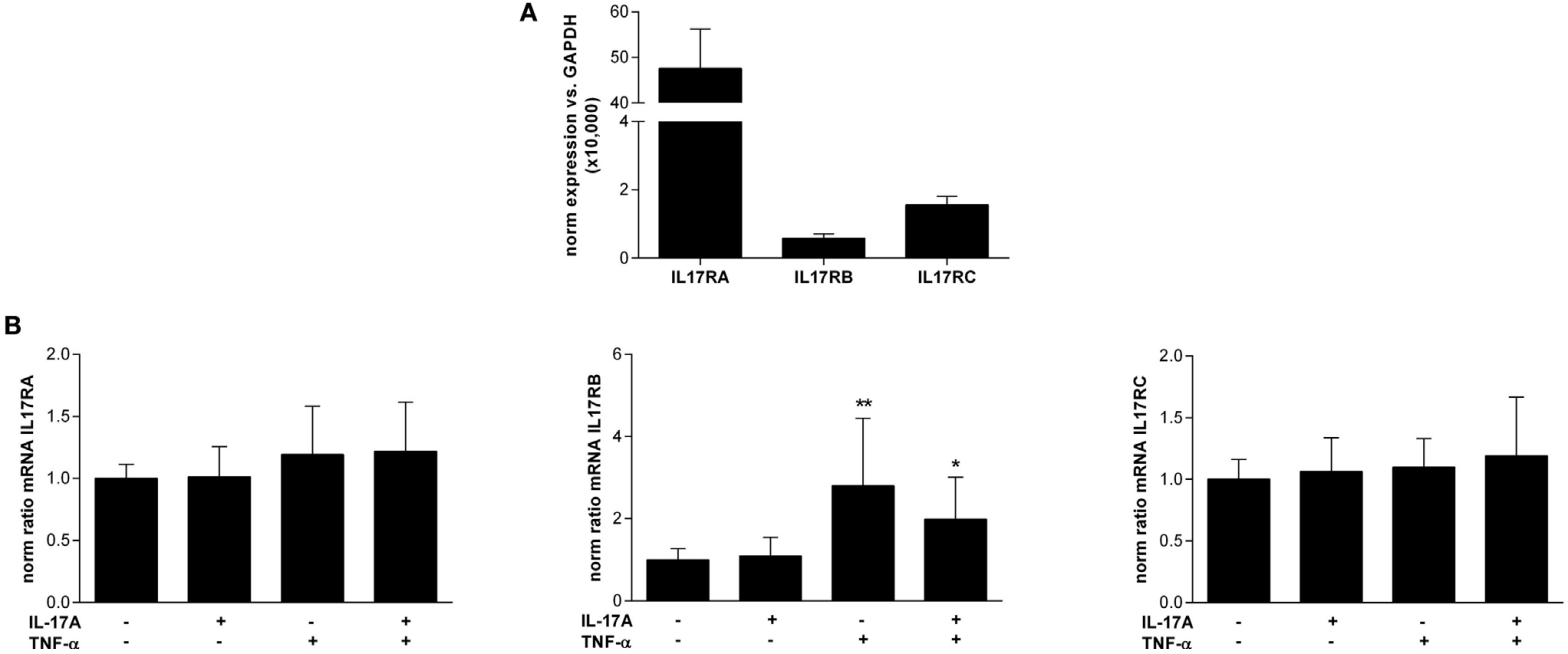
Expression of interleukin (IL)-17 family receptors IL-17RA, RB, and RC in synoviocytes. **(A)** RNA was extracted from different rheumatoid arthritis synoviocyte lines (*n* = 8) and the expression of IL17RA, RB, and RC was assessed by qRT-PCR. Results were normalized with GAPDH expression. **(B)** Cells were treated for 12 h with IL-17A (50 ng/mL) and/or tumor necrosis factor alpha (0.5 ng/mL) before RNA extraction. The expression of IL17RA, RB, and RC was assessed by qRT-PCR. *p < 0.05; **p < 0.01 (Mann–Whitney non-parametric test).

To study the mode of action of IL-25, the expression of IL-17 family receptors in resting synoviocytes was analyzed (Figure 4A). IL-17RB was the less expressed receptor of the family as its expression was 81-fold and 2.7-fold lower than that of IL17RA and IL17RC, respectively.

Then, the regulation of their expression after IL-17A and TNF-α treatment was investigated (Figure 4B). The expression of IL17RA and IL17RC receptor chains that form the IL-17A receptor complex was not affected by a treatment with IL-17A and/or TNF-α. In contrast, IL17RB expression was significantly increased after TNF-α treatment (2.8-fold increase compared to control without cytokine, *p* < 0.01), and to a lesser extent, after a treatment with a combination of IL-17A + TNF-α (2-fold increase compared to control without cytokine, *p* < 0.05).

### In Synoviocyte–PBMC Coculture, Early IL-17A Production Is Followed by Late IL-25 Production

To reproduce the cell–cell interactions seen at the site of inflammation, the expression and production kinetics for IL-6, IL-17A, and IL-25 were followed in a coculture model. Synoviocytes were cocultured with resting PBMCs or PHA-activated PBMCs. After 6, 12, and 24 h and 5 and 7 days, supernatants were collected for ELISA and mRNA were extracted for qRT-PCR analysis.

**Figure 5:**
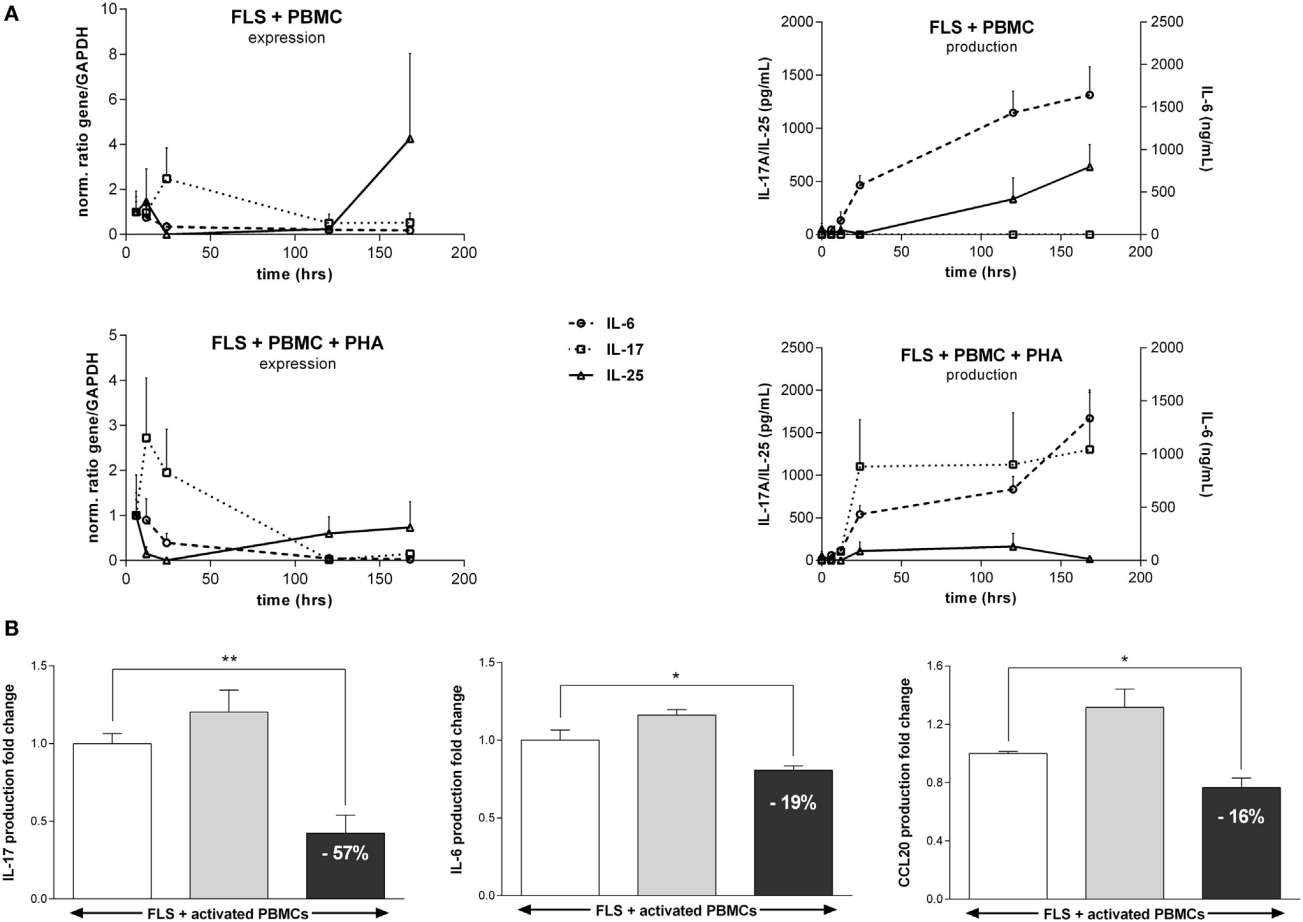
Kinetics of cytokine expression and production and effect of an interleukin (IL)-25 pretreatment in synoviocyte-peripheral blood mononuclear cell (PBMC) cocultures. **(A)** Synoviocytes were cocultured with PBMCs (1:5 ratio) activated or not. Coculture supernatants and RNA were collected at 6, 12, and 24 h and 5 and 7 days. IL-6, IL-17A, and IL-25 expression and production were quantified by qRT-PCR and enzyme-linked immunosorbent assay (ELISA), respectively. **(B)** Synoviocytes were cocultured with activated PBMCs (1:5 ratio). Either synoviocytes (light gray) or activated PBMCs (dark gray) were pretreated for 4 h with 50 ng/mL IL-25. After 24 h of coculture, supernatants were collected and the production of IL-17A, IL-6, and chemokine (C-C motif) ligand 20 was analyzed by ELISA. Results are expressed as a fold change of cocultured cells without pretreatment. *p < 0.05; **p < 0.01 (Mann–Whitney non-parametric test).

As shown in Figure 5A, IL-6 mRNA expression followed the same kinetics whether PBMCs were activated or not. It decreased quickly with a tendency of a more important decrease in cocultures with activated PBMCs than in cocultures with resting PBMCs (101-fold decrease vs. 58-fold decrease, respectively, non-significant). At the same time, IL-6 concentration in the medium increased over time.

When synoviocytes were cultured with non-activated PBMCs, there was an increased IL-17A mRNA expression during the first 24 h followed by a decrease. This early expression was not associated with any IL-17A protein production (Figure 5A). However, when synoviocytes were cocultured with activated PBMCs, IL-17A expression increased as soon as 12 h and then decreased. The IL-17A production also increased strongly up to 24 h (from 0 to 1,103 ± 954 pg/mL between 0 and 24 h, *p* = 0.03) and then to a lesser extent (from 1,103 ± 954 to 1,302 ± 1,407 pg/mL between 24 h and day 7, non-significant).

In the same conditions, IL-25 expression decreased during the first 24 h and then started increasing at late time points, starting from day 5, in both resting or activated PBMCs cocultures with a lower increase in cocultures with activated PBMCs. The IL-25 production also increased at late time points and was lower in coculture with activated PBMCs compared to coculture with resting PBMCs (637 ± 419 pg/mL in coculture with resting PBMCs vs. 18.7 ± 37 pg/mL in coculture with activated PBMCs; *p* = 0.03).

### IL-25 Inhibits IL-17A Production by PBMCs in Coculture

To better mimic the *in vivo* conditions, the effect of IL-25 pretreatment on a model of synoviocyte/PBMC coculture was analyzed. Synoviocytes were cocultured with activated PBMCs from healthy donors. Synoviocytes or PBMCs were pretreated with IL-25 for 4 h before the start of the coculture.

As shown in Figure 5, the pretreatment of synoviocytes showed no effect on IL-17A, IL-6, and CCL20 production in coculture. On the other hand, PBMCs pretreatment with IL-25 induced a 57% decrease in IL-17A production (*p* = 0.002). This decrease in IL-17A was associated with a lower but still significant decrease in IL-6 (19% decrease, *p* = 0.02) and CCL20 (16% decrease, *p* = 0.02) production.

### High IL-25 Levels in RA Patient Plasma

**Figure 6:**
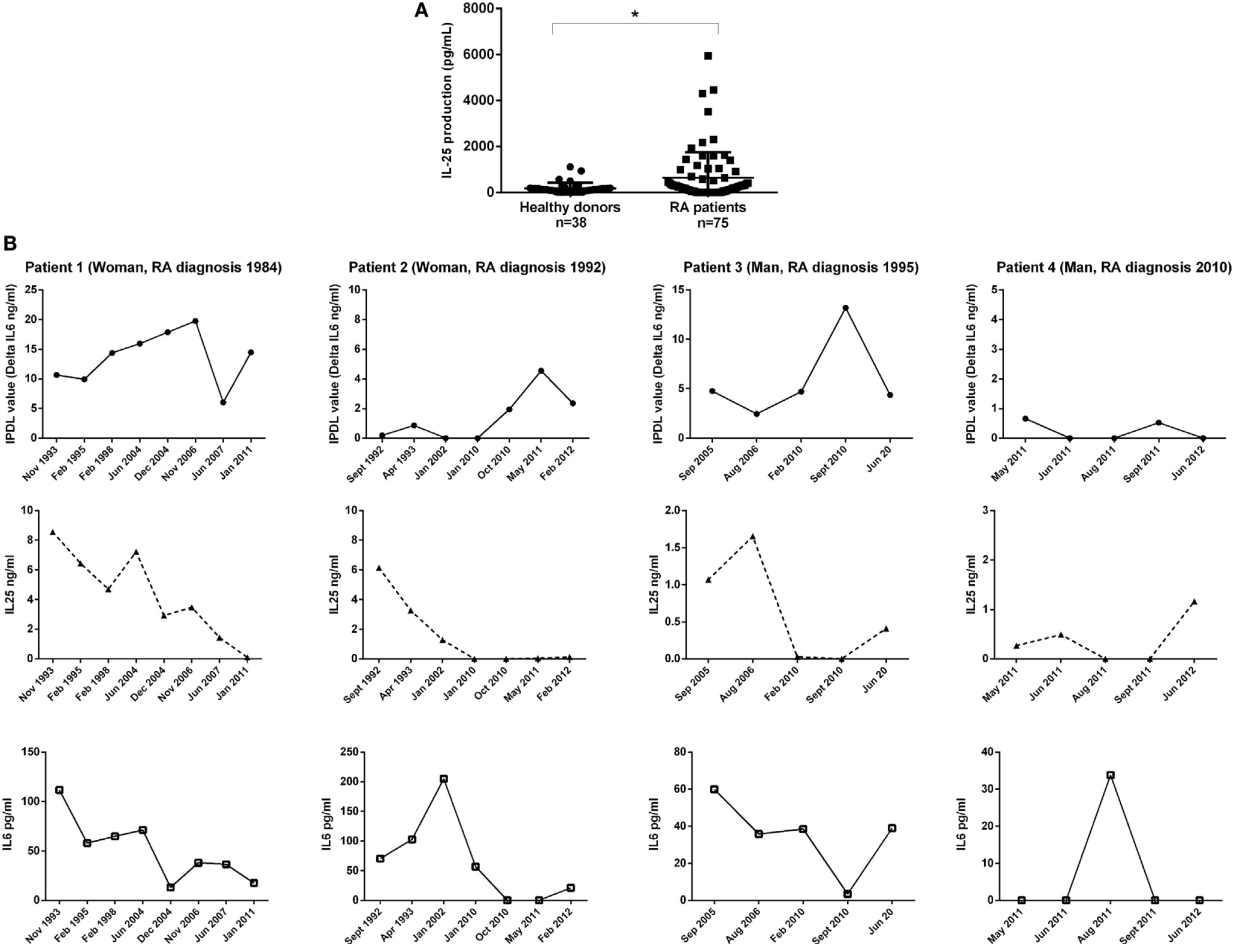
Quantification of interleukin (IL)-25, IL-17A, and IL-6 in rheumatoid arthritis (RA) patient plasma. IL-25 was quantified in the plasma of RA patients and healthy donors by enzyme-linked immunosorbent assay (ELISA) **(A)**; *p < 0.05 (Mann–Whitney non-parametric test). Long-term follow-up samples of four RA patients **(B)** were tested for IL-25 and IL-6 by ELISA. Bioactive IL-17A was assessed by a functional assay.

To expand the *in vitro* observations, levels of circulating IL-25 were measured by ELISA in the plasma of RA patients. As shown in Figure 6A, IL-25 levels detected in RA patients were significantly higher compared to healthy donors (641.1 ± 1110.7 pg/mL vs. 182.6 ± 240.1 pg/mL, *p* = 0.03) suggesting an association between IL-25 and RA. However, there was no correlation between IL-25 levels and disease severity (data not shown). Our observation also indicated that IL-25 was easy to detect using ELISA with levels ranging from hundreds to thousands picograms per milliliter, as opposed to IL-17A which is present at low levels by ELISA in the serum/plasma of RA patients.

### Balance between IL-17A and IL-25 Production in RA Patient Plasma

To study the kinetics of cytokine changes, IL-25 and IL-6 levels were measured by ELISA in a long-time follow-up of four different RA patients, for which samples were available during years of follow-up. Moreover, an original assay recently developed in our laboratory was used to measure IL-17A bioactivity (16). This functional assay is based on the sensitivity of endothelial cells to IL-17A. It allows the determination of IL-17A function in plasma, taking into consideration the contribution of activators (e.g., TNF-α acting in synergy with IL-17A) and inhibitors, namely, IL-25. In patients 2 and 3 (Figure 6B), with low inflammation (ESR = 10–15 mm/h), a modest elevation of IL-25 was detected as compared to patients 1 and 4 where high IL-25 levels were associated with high inflammation (ESR = 50–80 mm/h). This suggested that inflammation itself could represent a key activator of IL-25 production. In addition, there was an inverse correlation between bioactive IL-17A and IL-25 levels in three of four patients during the follow-up. Furthermore, the kinetic curve of IL-25 followed the same variations as those of circulating IL-6, suggesting that IL-25 could represent an attempt to control the contribution of IL-17A to inflammation. However, these increased levels of IL-25 are not high enough to control alone the function of circulating IL-17A. These preliminary observations that need to be extended are the first indication of a modified IL-17/IL-25 balance during chronic inflammation.

## Discussion

In this study, the presence and role of IL-25 in various RA cellular models was investigated and it was demonstrated that IL-25 has regulatory effects on IL-17A-induced inflammation, acting at the receptor level.

For the first time, it was showed that synoviocytes produce IL-25. Up until now, only hematopoietic (17–21), epithelial (22, 23), and endothelial cells (8, 24) were shown to produce IL-25. Moreover, the fact that synoviocyte IL-25 production occurred only after at least 5 days of culture was highlighted. At the same time, the levels of IL-6, which reflect the level of inflammation, showed opposite kinetics, increasing during the first days of culture and reaching a plateau after 5 days. Thus, IL-25 production occurred at a delayed time point after the induction of inflammation, in line with IL-25 acting as a negative regulator. These results extend the concept that IL-25 acts as a regulator of inflammation by controlling Th1 (25) and Th17 (8) responses and by inhibiting monocyte-derived inflammatory cytokines (26). These results are also in line with a recent study on the effect of IL-25 in RA, focusing on the interaction with the Th17 cell subset (10). In this study, IL-25 had an immunosuppressive role both *in vitro* and in a collagen-induced arthritis (CIA) model through a downregulation of the Th17 response. Here, we show that IL-25 also had an impact on synoviocytes reducing the effects of IL-17. IL-17 is critically involved in the chronicity of RA by inducing a long-term survival of synoviocytes (27).

In line with this first observation, when cells were treated with the combination of IL-17A and TNF-α, a decrease in IL-25 secretion was induced. TNF-α was already found to inhibit IL-25 secretion in *ex vivo* intestinal biopsies cultures (28). Here, the addition of IL-17A or TNF-α alone decreased IL-25 production but only their combination had a significant effect. Thus, the presence of pro-inflammatory factors not only increases the IL-6 secretion but also inhibits that of the inhibitor IL-25, resulting in a more potent inflammatory signal.

As IL-17A and IL-25 share the IL-17RA receptor subunit for signal transduction, it was interesting to determine if IL-25 can act as a receptor antagonist to inhibit IL-17A signal. Cells pretreated for 4 h with IL-25 were less responsive to IL-17A and, to a greater extent, to the combination of IL-17A and TNF-α. The modest effect observed on IL-17A signal can be explained by the weak IL-17RB expression compared to that of IL-17RA. Thus, IL-25 may only request a few IL17-RA chains, leaving several IL-17RA molecules available to transduce IL-17A signal. TNF-α has a regulatory effect by inducing the overexpression of IL-17RB, as already shown in synovial (29) and lung (30) fibroblasts where it was associated with an increased effect of IL-25. This induction of IL-17RB by TNF-α appears a way to limit inflammation though the inhibition by IL-25, and this may also explain the stronger inhibitory effect of IL-25 pretreatment on cells stimulated with both IL-17A and TNF-α.

The silencing of IL17R subunits showed the complexity of the interactions of each receptor chain in the response to IL-17A or IL-25. On the contrary, it raised questions on the specific role of IL17RB in response to IL-17A stimulation, as IL17RB silencing was shown to reduce synoviocyte sensitivity to IL-17A.

In a model to mimic the interactions between synoviocytes and mononuclear cells as at the inflammatory site, IL-25 could inhibit IL-17A production. Moreover, IL-25 decreased CCL20 and IL-6 production, two cytokines involved in Th17 cell recruitment (31) and differentiation (32), respectively. The pathogenic contribution of Th1 and Th17 cells and protective effects of Th2 cells have been described as a common feature of inflammatory diseases such as type 1 diabetes (7), multiple sclerosis (33), and RA (34). Conversely, IL-25 was shown to inhibit Th1 and Th17 cells and instead to promote the development of Th2 responses (8, 35). It was recently shown that treatment of PBMCs from RA patients with IL-25 inhibits IL-17A production (10). Here, these results were confirmed in a more relevant model using coculture between PBMCs and synoviocytes.

The study of the kinetics of cytokine production in the same coculture model showed a balance between IL-17A and IL-25 production. In our previous studies with the same model, IL-17A production required both cell–cell contact and PBMC activation (36, 37). In the same conditions, both IL-25 expression and production occurred at late time points, i.e., after 5 days. However, when PBMCs were activated and cocultured with synoviocytes, an early IL-17A expression and production followed by a late IL-25 expression and production were observed. Moreover, IL-25 production was reduced in the presence of IL-17A. This balance between IL-17A and IL-25 was already suggested in a mouse model of CIA with elevated IL-17A and low IL-25 levels at the onset and the opposite at a late stage (38).

Interleukin-25 expression has already been detected in the synovial fluid mononuclear cells of RA patients (39). The *in vitro* results were confirmed in clinical samples from RA patients. Not only the IL-25 levels were higher in RA patient plasma than in healthy controls, as already shown at a single time point (10), but a balance between IL-17A and IL-25 was also found in a kinetic study over many years. In a long-term follow-up of four RA patients from which serial samples covering several years were available, high levels of bioactive IL-17A were associated with low levels of IL-25 and *vice versa*. Furthermore, high plasma levels of IL-25 were associated with high IL-6 plasma levels. However, these plasma levels as well as the presence of local IL-25 in the joint do not appear high enough to control alone the IL-17A-driven inflammatory disease and to limit IL-17A activity.

In conclusion, *in vitro* and *in vivo* results provide evidence that IL-25 acts as a receptor antagonist competing at the receptor level with IL-17A. The involvement of IL-17RA in both IL-17A and IL-25 signal transduction may explain the differential effect of inhibitors of IL-17A and of IL-17RA (40, 41). These results are important to consider for the interpretation of the effect of IL-17A inhibition with anti-IL-17A vs. anti-IL-17RA antibodies. The two options differ from the inhibition of the anti-inflammatory effects of IL-25 when blocking the IL-17RA receptor chain.

## Ethics Approval

Ethics approval was done by ethics committee of the hospitals of Lyon.

## Patient Consent

Patient consent was obtained.

## Ethics Statement

Each individual signed an informed consent, and the protocol was approved by the committee for protection of persons participating in biomedical research under the number AC-2010-11-64.

## Author Contributions

FL: experiments and writing; NN-T: experiments; PM concept and writing.

## Conflict of Interest Statement

NN-T and PM hold a patent on the determination of bioactive IL-17. FL declares no conflict of interest.

## Abbreviations

IL, interleukin; RA, rheumatoid arthritis; PBMC, peripheral blood mononuclear cell; TNF-α, tumor necrosis factor alpha; CCL20, chemokine (C-C motif) ligand 20; IPDL, IL-17A pro-inflammatory dependent level.
